# Passive ankle movement increases cerebral blood oxygenation in the elderly: an experimental study

**DOI:** 10.1186/s12912-015-0066-x

**Published:** 2015-03-24

**Authors:** Sachiko Nagaya, Hisae Hayashi, Etsuko Fujimoto, Naoko Maruoka, Hiromitsu Kobayashi

**Affiliations:** Department of Nursing, Nagoya University Graduate School of Medicine, 1-1-20 Daiko-minami, Higashi-ku, Nagoya City, Aichi 461-8673 Japan; Department of Rehabilitation, Seijoh University, 2-172, Fukinodai, Tokai City, 476-8588 Japan; Department of Nursing, Ishikawa Prefectural Nursing University, 1-1 Gakuendai, Kahoku City, Ishikawa 929-1210 Japan

**Keywords:** Near infrared spectroscopy, Ankle exercise, Cerebral blood oxygenation

## Abstract

**Background:**

Ankle exercise has been proven to be an effective intervention to increase venous velocity. However, the efficacy of ankle exercise for improving cerebral circulation has not been determined. We hypothesized that ankle exercise in the supine position would be able to increase oxyhemoglobin levels measured at the forehead.

**Methods:**

Seventeen community-dwelling elderly women participated in this study. We recorded blood pressure, heart rate (HR), and oxyhemoglobin (OxyHb) levels from the participants in the supine position. Participants repeated ankle plantar flexion and dorsiflexion movements for 1 min. Two types of exercise were used: active movement and passive movement. We used two-way analysis of variance to assess the differences in mean arterial blood pressure (MAP), HR, and OxyHb between different exercises (active and passive) and times (before and after exercise).

**Results:**

The HR and MAP increased during active exercise but not during passive exercise. On the other hand, the levels of OxyHb measured at the forehead were elevated during both active and passive exercises. This increase lasted at least 1 min after exercise. There was no significant difference between active and passive exercise with regard to OxyHb; however, a significant difference was observed between before and after exercise (*p* < 0.05, η^2^_G_ = 0.153).

**Conclusions:**

The physiological response of OxyHb to ankle exercise was different from that of the other cardiovascular functions. Both active and passive ankle exercises were able to increase cerebral blood oxygenation, whereas the other cardiovascular functions did not respond to passive exercise.

## Background

A postural change from supine to standing shifts approximately 500–700 ml of blood toward the lower body. This fluid shift causes a temporal decrease in venous return to the heart and a reduction in cardiac output, thereby inducing transient arterial hypotension. When blood pressure falls, the baroreceptor reflex works immediately. This reflex elicits an increase in the heart rate (HR) and total peripheral resistance to maintain a normal blood pressure level [1]. However, it takes a few seconds to restore the blood pressure. Therefore, a postural change sometimes induces temporal hypotension.

A postural change does not generally cause severe fluctuations in blood pressure for healthy people. However, some people have symptoms related to hypotension, such as dizziness, syncope, and falls, a condition that is well known as “orthostatic hypotension”. The prevalence of orthostatic hypotension is observed in the elderly because baroreceptor sensitivity decreases with aging [2]. It has been reported that orthostatic hypotension contributes to falls in the elderly [3] and is a significant risk factor for cardiovascular disease [4]. In hospitalized elderly people, orthostatic hypotension is a relatively common condition [5-7]; therefore, nurses are routinely required to pay attention to its signs. Management of orthostatic hypotension is thought to be an important issue.

Several pharmacological and nonpharmacological approaches to prevent orthostatic hypotension have been proposed. Nonpharmacological approaches are better for the first line of treatment because they do not have adverse effects. Compression garments or tightly fitting body stockings are useful for improving orthostatic hypotension because they can reduce the venous capacitance bed [8]. However, it seems difficult for elderly patients to be able to put on tight fitting stocking by themselves. Active ankle exercises have been proven to be an effective intervention to increase venous velocity by squeezing accumulated blood from the lower part of the body [9-11]. Plantar and dorsiflexion ankle movements involve movements of the gastrocnemius and soleus muscles. When the ankle joint moves, these muscles can work as the muscle pump. Leg muscle contractions lead to mechanical compression of the venous vascular beds. Considering these mechanisms, ankle exercise may be able to prevent the hemodynamic changes induced by postural change.

The fundamental cause of orthostatic hypotension symptoms is impaired cerebral hemodynamics [12]. Therefore, measurement of cerebral hemodynamics can provide information about the direct cause of symptoms associated with postural change. However, little attention has been given to the effect of ankle exercise on changes in cerebral hemodynamics.

Several devices have been developed to estimate cerebral hemodynamics. Transcranial Doppler (TCD) and near infrared spectroscopy (NIRS) are the major continuous and noninvasive methods available to measure cerebral hemodynamics. They measure cerebral hemodynamics from different aspects. The TCD method measures changes in blood flow velocity in large cerebral arteries such as the middle cerebral artery. On the other hand, NIRS measures changes in cerebral oxygenation in the cerebral cortical tissue using near infrared light. Although these techniques are different, previous studies have revealed that both blood flow velocity and cerebral oxygenation showed similar changes with orthostatic stress [13-15]. From these studies, it can be concluded that both techniques are suitable to study the estimated changes in cerebral hemodynamics. However, TCD requires advanced techniques to create accurate images. Therefore, NIRS may be the better technique for measuring cerebral hemodynamics associated with ankle exercise.

Ankle exercises are simple to execute, even for elderly patients. Therefore, if these exercises are proven to be effective in maintaining cerebral hemodynamics, it can be presumed that they may develop into a new intervention for preventing the symptoms related to postural change. This could be particularly helpful for elderly people. In addition, if a beneficial effect of passive ankle exercise is revealed, it could be useful for immobilized patients because nurses will be able to use it to enhance cerebral blood oxygenation.

The present study aimed to reveal the initial phase of changes in cerebral oxygenation induced by active or passive ankle exercise.

## Methods

### Participants

We recruited community-dwelling elderly volunteers based on the following criteria: (i) age 65 years and older and (ii) absence of cardiac arrhythmias and/or impaired motor function. They voluntarily chose to participate in this study; therefore, there were no participants who refused the experiments.

The study consisted of 17 community-dwelling elderly women aged 69–83 years (74 years on average). Their mean height, weight, and body mass index were 150.2 ± 4.3 cm, 52.9 ± 5.7 kg, and 23.4 ± 2.2 kg/m^2^, respectively. Prior to the study, the participants were instructed to abstain from alcohol for 12 h, not to eat a meal within 90 min, and to get enough sleep.

### Experimental procedures

The experiments were conducted in a climatic chamber to maintain stable environmental conditions. We maintained the room temperature at 25 °C with 50% relative humidity. Before the measurements, we explained the experimental protocols in detail to the participants. In addition, the participants practiced the ankle exercises with the researcher.

**Figure 1:**
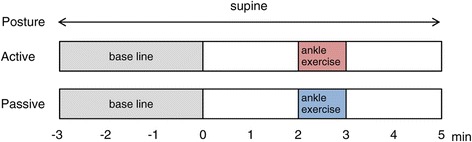
**Schematic representation of the experimental protocol.** The ankle exercises began at 2 min. The physiological responses were monitored throughout the experiment.

The protocols are illustrated in Figure 1. Prior to the measurement of oxyhemoglobin (OxyHb), we recorded the OxyHb of each subject for 3 min to obtain a baseline value. After the baseline had been measured, participants maintained a supine position for 2 min. Then, each participant performed active or passive ankle movements. For active ankle exercise, the participants performed plantar flexion and dorsiflexion movements of the ankle joint alternately for 1 min. For passive ankle exercise, the researcher moved the participant’s ankle in a similar manner as that described for active exercise. The pace of ankle movements was 60 times/min for both active and passive exercises. We determined the manner of exercise by referring to previous studies [11,16]. After the exercise, the participants rested again. The aim of this study was to estimate the early changes in cerebral OxyHb after ankle exercise. Therefore, we set the measurement periods after the ankle exercises to 2 min.

Throughout the experiments, including the exercise period, the participants lay supine on a bed. Each participant was evaluated under both active and passive exercise conditions. The order of the conditions was counterbalanced among the participants to avoid carryover effects.

### Measurements of physiological responses

We measured cerebral blood oxygenation by NIRS (NIRO-120; Hamamatsu Photonics, Shizuoka, Japan) at a sampling rate of 2 Hz. Near infrared light can penetrate biological tissue and cause changes in OxyHb and deoxygenated hemoglobin concentration. An optode of NIRO-120 was attached on the left side of forehead of the participants during the experiment. The left side of forehead was selected as the measurement region because previous studies using NIRS did not show any difference between the right and left forehead during moderate exercise [17]. The value of OxyHb was converted by calculating the difference from the 3 min baseline recording.

We monitored the HR and systolic and diastolic blood pressures (SBP and DBP, respectively) using a BP-608 Evolution II (Omron Colin, Tokyo, Japan), which continuously captured these cardiovascular signals (beat-by-beat). The mean arterial blood pressure (MAP) was calculated by adding one-third of the pulse pressure to the DBP. The HR and MAP recordings were linearly interpolated into 2-Hz equidistant signals to synchronize with the NIRS recording.

### Statistical analysis

We assembled the physiological signals (HR, MAP, and OxyHb) of the 17 participants to indicate average continuous responses to the exercise. Moreover, the signals before exercise (from 1 min to 2 min, Figure 1) and after exercise (from 3 min to 4 min, Figure 1) were individually averaged for 1 min for two-way repeated measures analysis of variance (ANOVA). Hereafter, the factor related to the type of exercise (active or passive) is expressed as the TYPE factor and the factor related to time (before or after) is expressed as the TIME factor. We set statistical significance at *p* < 0.05. Generalized eta squared (η^2^_G_) values were presented as the effect size of each factor. The η^2^_G_ values can be interpreted in the same manner as η^2^ values; therefore, η^2^_G_ values of 0.01, 0.059, and 0.138 were interpreted as small, medium, and large effects, respectively [18]. Statistical tests were performed by R 3.0.3 for Windows.

### Ethical considerations

The study protocol was approved by the Ethics Committee of Nagoya University Graduate School of Medicine, Japan.

The purpose and methods of the study were stated to the participants, and we obtained written informed consent from those who agreed to participate. Participants were informed that they could refuse to participate or withdraw from the study at any time. Before the start of the experiment, we checked participants’ physical conditions (such as the presence of knee pain) carefully. Participants could take breaks between each measurement at which they were asked whether they wanted to discontinue the measurements.

## Results

**Figure 2:**
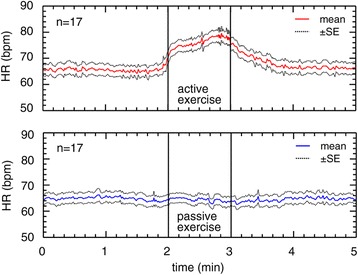
**Heart rate responses to active and passive ankle exercises (n = 17).** Data are given as a mean ± standard error (SE). A solid line indicates the mean heart rate (HR). A dashed line indicates the SE. The ankle exercises were performed between 2 and 3 min.

All the participants completed the experimental protocol. Figure 2 demonstrates the time course of the HR responses to active and passive ankle exercises. With the active ankle exercise, HR was markedly elevated during exercise (2–3 min), whereas there was no change during the passive ankle exercise. At the end of the active ankle exercise, the HR shifted from increasing to decreasing and then, HR recovered to baseline level for 1 min after the active exercise. Passive exercise did not change HR throughout the experimental period.

**Figure 3:**
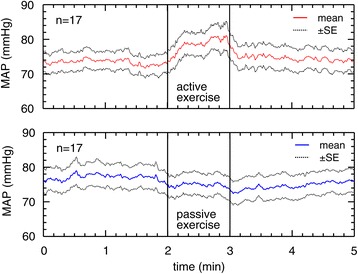
**Mean arterial blood pressure responses to active and passive ankle exercises (n = 17).** The data are given as a mean ± standard error (SE). A solid line indicates the mean arterial blood pressure (MAP). A dashed line indicates the SE.

Figure 3 illustrates the time-course response of MAP to active and passive ankle exercises. The MAP increased only during active exercise. In passive ankle exercise, there was no change in MAP accompanying plantar flexion and dorsiflexion ankle movements.

**Figure 4:**
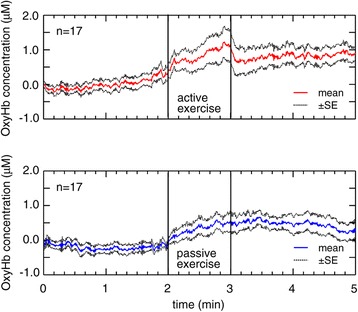
**Oxyhemoglobin responses to active and passive ankle exercises (n = 17).** The data are given as a mean ± standard error (SE). A solid line indicates the oxyhemoglobin (OxyHb) level. A dashed line indicates the SE.

Figure 4 shows the time-course response of cerebral oxygenation to ankle exercise in the supine position. A progressive rise in OxyHb measured at the forehead was demonstrated during both active and passive exercises. After completing active exercise, OxyHb immediately decreased. Thereafter, it showed an almost parallel change and maintained a high level in comparison with the values from before the exercise. Passive exercise had the same tendency as active exercise and OxyHb showed a gradual decrease after passive exercise.

**Figure 5:**
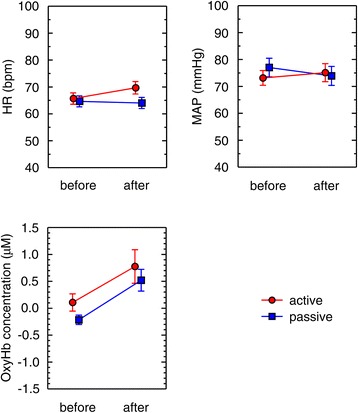
**Effects of active and passive ankle exercises on 1-minute averages of heart rate, mean arterial blood pressure, and oxyhemoglobin (n = 17).** The data are given as a mean ± standard error (SE). Circles: mean value of active ankle exercise; squares: mean value of passive ankle exercise. Before: each value represents the mean value from 1 min to 2 min; after: each value represents the mean value from 3 min to 4 min.

Figure 5 shows the effects of the exercise on the 1-min averages of the HR, MAP, and OxyHb. In Figure 5, the data of before exercise was obtained from 1 min to 2 min and the data of after exercise was obtained from 3 min to 4 min. Results of the two-way ANOVA are summarized in Table 1.

**Table 1:** **Results of ANOVA for effects of the TYPE factor and TIME factor on ankle exercises**

**Factor**	**HR**		**MAP**		**OxyHb**	
	**(bpm)**		**(mmHg)**		**concentration(μM)**	
	*p*	η^2^_G_	*p*	η^2^_G_	*p*	η^2^_G_
TYPE	< 0.01**	0.035	0.56	0.003	0.13	0.030
TIME	< 0.01**	0.011	0.54	0.001	< 0.05*	0.153
TYPE*TIME	< 0.01**	0.020	< 0.01**	0.009	0.78	< 0.001

*, *p* < 0.05; **, *p* < 0.01.

η^2^_G_ : effect size for each factor.

The HR and MAP showed different tendencies depending on the type of exercise. For HR, both TYPE and TIME factors were significant (*p* < 0.01, η^2^_G_ = 0.035 and *p* < 0.01, η^2^_G_ = 0.011, respectively). In addition, a significant interaction (*p* < 0.01, η^2^_G_ = 0.020) was observed with HR. For MAP, neither TYPE nor TIME factors showed statistical significance. There was a significant interaction effect with MAP (*p* < 0.01, η^2^_G_ = 0.009).

On the other hand, active and passive exercises showed parallel responses with regard to OxyHb. For OxyHb, no significant effect was observed on the TYPE factor, whereas a significant effect was observed on the TIME factor (*p* < 0.05, η^2^_G_ = 0.153). The ANOVA did not show an interaction effect with OxyHb.

In summary, we found different effects of active and passive ankle exercises on HR and MAP; however, both types of exercise increased OxyHb levels measured at the forehead.

## Discussion

### Responses of HR and MAP to ankle exercise

Previous studies have reported that cardiovascular responses to exercise depend on the type of exercise. Active limb exercise increased blood pressure significantly; however, it was not observed during passive exercise [19]. Active elbow exercise was found to increase HR; however, passive elbow exercise did not show the same effect [20]. Liang et al. [21] compared voluntary and involuntary exercises. They defined voluntary exercises as those performed by the participants and involuntary exercises as those evoked by electrical muscle stimulation. They found that both HR and MAP increased with voluntary ankle dorsiflexion whereas there were no significant changes in HR and MAP with involuntary exercise. In our study, increases in HR and MAP over time occurred only during active exercise and not passive exercise. These results are in line with those of previous studies [19-21]. The difference in the responses between active and passive exercise could be attributed to a difference in the workload of the exercise.

### Responses of cerebral hemodynamics to ankle exercise

Active ankle exercise increases blood velocity in the popliteal [11] and femoral veins [9,10] because it can elicit the function of the skeletal muscle pump. The skeletal muscle pump plays an important role in promoting venous return. As a result, cardiac filling pressure and cardiac output increase, ultimately leading to an increase in cerebral perfusion [22]. In this study, both active and passive ankle exercises increased cerebral OxyHb. With regard to different types of exercise (for example, hand or lower limb), previous studies have reported that both active and passive exercises affect cerebral blood velocity [19,23]. These previous results agree with the results of our study, which were measured by NIRS. Both active and passive ankle exercises appear to produce similar types of mechanical stimuli, although differences in intensity exist. Thus, stimuli from either active or passive ankle exercise could contribute to increased cerebral oxygenation.

NIRS has been used as a method of measuring the brain functions associated with exercise. It has been reported that OxyHb is elevated by motor imagery [24]. In our study, the value of OxyHb before exercise seemed to be different between active and passive exercise (Figure 5). This difference in OxyHb may represent brain activity that was evoked by motor imagery in the participant’s mind in preparation for active exercise. However, motor imagery effects were considered relatively small compared with that of ankle exercise because the effect of motor imagery is short-lived [24].

Postural change causes some degree of drop in blood pressure because fluids in the body shift under the influence of gravity. Activation of the baroreceptor reflex generally controls HR and total peripheral resistance to maintain adequate pressure [25]. However, baroreceptors need a little time to work. Gravitational fluid shifts also have an effect on cerebral circulation. Therefore, some people have light-headedness or dizziness when baroreceptor reflection cannot fully compensate for changes in cerebral circulation. This phenomenon has been documented in studies using NIRS. Previous studies have shown significant declines in frontal cortical blood oxygenation in patients with autonomic failure [26] and healthy elderly people [27,28] when they stand up. From these reports, we know that maintaining cerebral oxygenation is essential for preventing symptoms associated with postural change.

Galizia et al. [29] have revealed that supine leg exercises are effective in mitigating the initial drop in blood pressure in people with orthostatic hypotension; however, their study did not investigate cerebral oxygenation. We confirmed that ankle exercise in the supine position can increase OxyHb measured at the forehead and that it lasts at least 1 min after exercise. Hemodynamic changes following postural changes commonly occur in the short period immediately after moving [30-32]. The increased duration of the change in OxyHb evoked by ankle exercise is considered sufficient to span the timing of the initial change in hemodynamics induced by postural changes. Therefore, we hypothesize that ankle exercises may be useful for preventing symptoms associated with postural change.

We emphasize the benefits of passive exercise. Time-course response of passive ankle exercise showed that it could increase OxyHb without changes in HR and MAP (Figures 2, 3 and 4). In passive exercise, participants did not voluntarily move their ankles; therefore, their cardiovascular systems were not required to adjust HR and MAP to meet the muscle demands. However, the venous capacitance bed was compressed by the passive stimuli; consequently, cerebral OxyHb was increased. These responses to passive exercise could be especially beneficial for patients who have cardiovascular problems. In addition, passive ankle exercise may be beneficial for immobile patients because nurses can passively promote their cerebral oxygenation.

### Limitations of this study

There are a number of limitations to this study. We recruited volunteer participants and only women participated in this study. A cautious interpretation may be recommended when to apply the results beyond the gender difference. The small sample size is another limitation of this study. We did find significant changes in OxyHb associated with ankle exercises from this small sample size. Active ankle exercise seemed to have more effect than passive exercise (Figure 5), however, it was not statistically significant. A larger sample size may be able to reveal the difference of effect between active and passive exercise.

This study has revealed that ankle exercise has a large effect size on cerebral OxyHb. The association between statistical significance (a small *p*-value) and clinical significance has been discussed for years [33]. Although statistical and clinical significances are not completely identical, the effect size is considered a more appropriate indicator to estimate the clinical significance rather than a *p*-value. However, this study was an experimental study, not a clinical intervention study. Therefore, further study will be needed to validate the effects of ankle exercise on patients.

## Conclusions

We investigated the effects of ankle exercise on HR, MAP, and OxyHb. The physiological response of OxyHb to ankle exercise was different from that of the other cardiovascular functions. Both active and passive ankle exercises increased cerebral blood oxygenation, although the other cardiovascular functions showed no response to passive exercise.

## Abbreviations

ANOVA: Analysis of variance
DBP: Diastolic blood pressure
HR: Heart rate
MAP: Mean arterial pressure
NIRS: Near infrared spectroscopy
OxyHb: Oxyhemoglobin
SBP: Systolic blood pressure
SE: Standard error
TCD: Transcranial Doppler
η^2^_G_: generalized eta squared
